# Editorial: Mathematical Modeling of Endocrine Systems

**DOI:** 10.3389/fendo.2021.789386

**Published:** 2021-11-04

**Authors:** Darko Stefanovski, Giovanni Pacini, Ray C. Boston

**Affiliations:** ^1^Clinical Studies-New Bolton Center, School of Veterinary Medicine, University of Pennsylvania, Philadelphia, PA, United States; ^2^Institute of Neurosciences, Italian National Research Council, Padova, Italy; ^3^University of Pennsylvania, Philadelphia, PA, United States

**Keywords:** mathematical modeling and simulation, selenium, free fatty acid (FA) metabolism, glucose metabolism, glucagon kinetics, ovarian simulations, insulin secretion, minimal model of glucose metabolism

Mathematical modeling is a process for formulating a set of equations to simultaneously represent a system’s structure and behavior. While in the majority of cases, the equations of the mathematical model are non-linear and designated using ordinary differential equations (ODEs), this does not exclude either models that are as simple as a single linear equation or even a more complex set of hundreds of partial differential equations (PDEs). A “system” that is subjected to modeling can include several organ systems or be as limited to focusing only on a specific interaction between cells. Sometimes the model can even focus on a single cell, or on an entire cell line.

Commonly, in scientific endeavors, the inception of models starts with observations: or more specifically, with a set of samples taken over time from a single entity (subject, animal, tissue sample, or cells) or perhaps following the system’s response to a perturbation. The aim is inevitably to build a mathematical account that responds to the observed data of the underlying biological system. Thus, models offer insights into the mechanisms and signal transduction pathways, and provide the bedrock for hypothesis-generating research. Furthermore, the parameters of the model may conveniently serve as biomarkers of specific biological mechanisms, or of patho-physiological states.

We are very enthusiastic to have in this special modeling edition a vibrant and informative historical account of the development of one of the most successful and widely used mathematical models of a biological system: i.e., the Minimal Model of Glucose Kinetics. The original developer, Dr. Richard N. Bergman, outlines the merits of the model, which indeed the great majority of the investigators from the listed authors of this Research Topic have, for more than 15 years, used in their metabolic research projects. Some have also been responsible (the three editors included) for the release of automated computational tools to perform frequently sampled IVGTT data analyses for the rapid, and precise, estimation of Insulin Sensitivity and Glucose Effectiveness.

As the title of this Research Topic suggests, the aim of this collection of papers is to provide interesting and novel information on various facets of mathematical modeling of endocrine systems. Four articles focus on various aspects of mathematical modeling of endocrine control of glucose metabolism. Morettini et al. investigate glucagon kinetics and its relationship with insulin during an oral glucose challenge (OGTT); using the output from a simple model, they are able to assess pancreatic alpha-cell sensitivity to insulin. Schiavon et al. describe the issues encountered with modeling insulin secretion using a model of C-peptide kinetics in post gastric bypass patients with Type 2 Diabetes. Ward et al. describe the modifications needed in mathematical models of insulin secretion/kinetics, and in glucose metabolism, to use data obtained with islet transplant recipients with Type 1 Diabetes. The work by Hu et al. (D’Argenio’s group) focuses on the successful integration of mathematical models, and hierarchical statistical models, to obtain more accurate population estimates of Glucose Effectiveness, which – together with Insulin Sensitivity – characterizes the glucose dynamics during glucose challenges.

An article from Boston’s group delves into problems linked to automatically, and accurately quantifying the manifest features of lactate infusions: these are essential to gain insights into the persistence of both exogenous and endogenous lactate in conjunction with such challenges.

Work by Stefanovski et al. describes the development of a novel model of whole-body FFA kinetics, and this enables the estimation of insulin action in regard to adipose tissue. Indeed, the model actually quantifies the ability of insulin to rapidly suppress lipolysis during the frequently sampled IVGTT.

The article from Patterson et al. covers the development of a model of Selenium (Se), in regard to both endocrine, and to immune, systems. The report estimates the kinetics of Se before and after 2 years of Se administration.

The work of Fischer et al. outlines previously developed models of the menstrual cycle that are capable of simulating control administrations, including, for example, ovarian contraception pills, and GnRH analogs. These can then be used for *in-silico* experiments that may help to improve ovarian stimulation.

We sincerely hope that the contributions outlined above will show how your own future interests in applying mathematical modeling methods might help advance new challenges in kinetic investigations for you. Looking forward to these efforts, we would also like to remind you of the importance of using mathematical models *per se …* while simulation and additional *in-vivo* studies can provide evidence of the validity and repetability of a model, it is the continuous use of models by the general scientific community that will assure their reliability and robustness.

## Author Contributions

All authors listed have made a substantial, direct, and intellectual contribution to the work and approved it for publication.

## Conflict of Interest

The authors declare that the research was conducted in the absence of any commercial or financial relationships that could be construed as a potential conflict of interest.

## Publisher’s Note

All claims expressed in this article are solely those of the authors and do not necessarily represent those of their affiliated organizations, or those of the publisher, the editors and the reviewers. Any product that may be evaluated in this article, or claim that may be made by its manufacturer, is not guaranteed or endorsed by the publisher.

